# Evaluating the efficacy and safety of combined microneedling therapy versus topical Minoxidil in androgenetic alopecia: a systematic review and meta-analysis

**DOI:** 10.1007/s00403-025-04032-1

**Published:** 2025-03-08

**Authors:** Khalid M. A. Ahmed, Yasmeena Abdelall Kozaa, Mohammad T. Abuawwad, Alaa I. Al-Najdawi, Yomna W. Mahmoud, Ahmed M. Ahmed, Mohammad J. J. Taha, Tamara Fadhli, Angeliki Giannopoulou

**Affiliations:** 1 Leeds Institute of Medical Education, University of Leeds, Woodhouse, Leeds, LS2 9JT UK; 2 Mansoura Manchester Program for Medical Education, Faculty of Medicine, Mansoura University, Mansoura, Egypt; 3 Kasr Al Ainy School of Medicine, Cairo University, Al Giza, Egypt; 4 Faculty of Medicine, The University of Jordan, Amman, Jordan; 5 Department of Dermatology, West Hertfordshire Teaching Hospitals NHS Trust, London, UK; 6 St. John’s Institute of Dermatology, Guy’s and St Thomas’ NHS Foundation Trust, London, UK

**Keywords:** Androgenetic alopecia, Hair count, Hair diameter, Depth, Diameter, Rolling microneedling, Electrodynamic microneedling, Microneedling

## Abstract

This study evaluates the efficacy and safety of the combined microneedling (CMNT) with minoxidil versus minoxidil monotherapy for the treatment of androgenetic alopecia (AGA), with a focus on the impact of microneedling parameters on treatment outcomes. We conducted a systematic review and meta-analysis (PROSPERO: CRD42024594487) of randomized controlled trials (RCTs) comparing CMNT versus minoxidil alone for AGA, following PRISMA guidelines. A comprehensive search across six databases was performed up to September 8, 2024. We identified 12 RCTs involving 631 AGA patients, with a total of 11 RCTs included in the meta-analyses. CMNT significantly improved hair count compared to minoxidil monotherapy (SMD 1.32, 95% CI 0.73–1.92, *p* < 0.01), with substantial heterogeneity (I² = 88%, *p* < 0.01). Subgroup analyses indicated no significant effect of microneedling (MN) depth (≤ 1 mm vs. >1 mm), duration (≤ 12 weeks vs. >12 weeks), or technique(device) (electrodynamic vs. rolling) on hair count outcomes. Additionally, A meta-analysis of six RCTs demonstrated a significant improvement in hair diameter with CMNT (SMD 0.34, 95% CI 0.11–0.58; *p* < 0.01), with no observed heterogeneity (I² = 0%). Investigators and patient’s self-assessment scores were also improved. Adverse events were more frequent with CMNT (74 vs. 59 events), however they were generally considered mild or self-limiting. CMNT significantly enhances hair count and diameter in AGA patients with mild adverse events. MN parameters including depth, duration, and technique variations did not significantly affect hair count outcome, suggesting microneedling as a promising adjunctive AGA treatment.

**Supplementary Information:**

The online version contains supplementary material available at 10.1007/s00403-025-04032-1.

## Introduction

Androgenetic alopecia (AGA) is a common type of hair loss that affects both men and women, typically starting after puberty and progressively increasing with age [1]. It has been proven that AGA negatively affects patients’ quality of life, frequently leading to psychological distress, including depression, anxiety, low self-esteem, and social withdrawal [2]. AGA is characterized by follicular miniaturization attributed to dihydrotestosterone (DHT), which disrupts the Wnt/β-catenin signaling pathway, increases the expression of transforming growth factor β1, and induces apoptosis in dermal papilla and epithelial cells [3, 4]. This cascade eventually causes hair follicle miniaturization by reducing dermal papillae cell clusters, and shortening the anagen phase [5, 6].

The primary goal of AGA therapy is to prevent follicular miniaturization and stabilize hair loss [7]. Currently, Minoxidil and Finasteride are the only FDA-approved medications for treating AGA [8, 9]. However, these treatments have limitations, including inconsistent response rates and the poor transdermal absorption of topical minoxidil [8]. Additional non-surgical therapies, such as dutasteride, spironolactone, platelet-rich plasma (PRP), and low-level laser therapy (LLLT), have also been employed [10].

Microneedling (MN) is an emerging, minimally invasive procedure, that has been used as an adjunctive treatment for AGA. By creating micro-injuries in the skin, MN stimulates angiogenesis, collagen production, and the initiation of a new anagen phase. This process activates hair follicle stem cells and stimulate the release of platelet-derived growth factor and vascular endothelial growth factor [11–13]. Additionally, MN has been demonstrated to reduce perifollicular fibrosis, which frequently develops in the late stages of AGA and can compromise the effectiveness of topical and systemic therapies [14]. By creating microchannels in the stratum corneum, MN improves transdermal drug delivery as an adjuvant therapy. This increases the rate of absorption and accelerates the onset of action for topical medications such as minoxidil [15, 16].

Several RCTs have examined the efficacy of the combined microneedling with topical minoxidil (CMNT) versus minoxidil monotherapy. These studies vary in microneedling depth, duration, and technique (device). The objective of this study is to present comprehensive evidence on the efficacy and safety of CMNT compared to minoxidil monotherapy, with a focus on the impact of microneedling parameters such as depth, duration, and technique on treatment effectiveness.

## Methods

This systematic review has been reported according to the preferred reporting items of systematic reviews and meta-analysis (PRISMA statement) [17]. The review protocol has been registered on PROSPERO with the registration ID: CRD42024594487.

### Criteria of included studies

Studies satisfying the following inclusion criteria were included in the systematic review:

#### Study design

Randomized controlled trials (RCTs).

#### Population

Participants aged 18 years or older diagnosed with androgenetic alopecia (AGA) were included.

#### Intervention

The combined microneedling with topical minoxidil (CMNT).

#### Comparator

Minoxidil monotherapy.

#### Outcome

Eligible outcomes included the primary outcome of assessing the efficacy of CMNT versus minoxidil monotherapy, measured by the mean change in overall hair count and hair diameter from baseline to post-treatment, as well as investigator’s and patient’s assessments of hair regrowth. The secondary outcome focused on safety, determined by the number of patients reporting adverse events in each group.

**Fig. 1:**
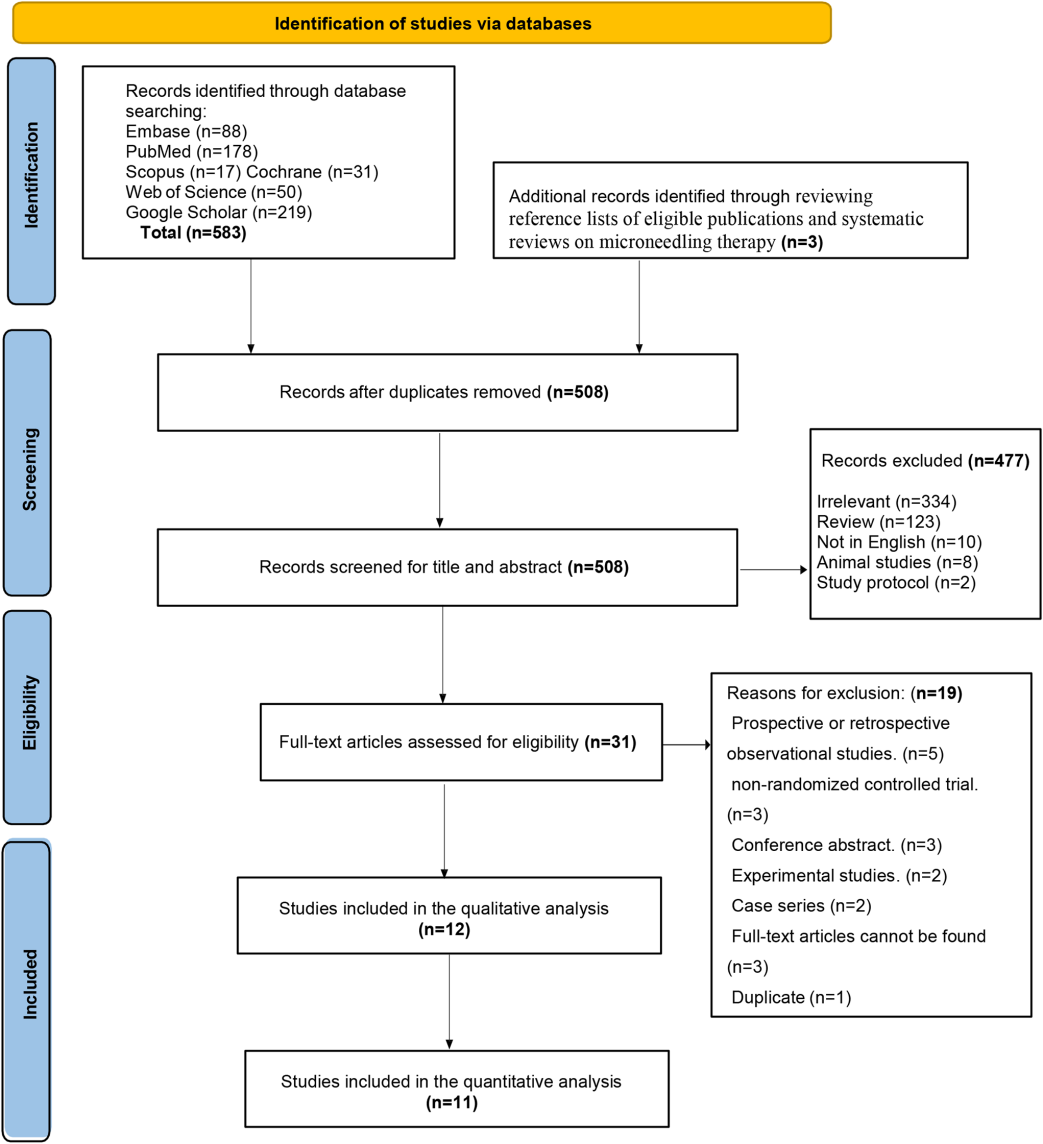
PRISMA flow diagram illustrating the study selection process for the systematic review and meta-analysis

Only English-language studies published in peer-reviewed journals were included. Excluded studies were: (1) non-RCTs, (2) animal studies, (3) those lacking comparisons between combined microneedling therapy and minoxidil monotherapy, (4) non-peer-reviewed publications (e.g., conference abstracts), (5) non-English studies, and (6) studies with insufficient or inaccessible data. The PRISMA flow diagram of the study selection process is shown in (Fig. 1).

### Literature search and keywords

An electronic search was conducted in PubMed, Cochrane Library, Embase, Scopus, Web of Science, and Google Scholar from inception to September 8, 2024. MESH keywords and Boolean operators (AND, OR) were applied. The detailed search strategy is in the supplementary file. A ‘snowball’ search was also performed by reviewing reference lists of eligible publications and systematic reviews on microneedling therapy.

### Screening and study selection process

We used Rayyan for semi-automated screening of the literature search results [18]. Studies were screened in two phases. The first phase was title/abstract screening for potential clinical studies. In the second phase, we retrieved the full-text articles of the selected abstracts for further eligibility screening.

### Data extraction

Data were extracted using a piloted online Google form. Two independent reviewers collected data separately, with discrepancies resolved through discussion with a senior reviewer. Extracted data were categorized into six domains: (1) study characteristics, (2) population characteristics, (3) intervention details (needle diameter, depth, technique, interval, additional topical treatments, minoxidil dose/frequency, and follow-up duration), (4) efficacy outcomes, (5) adverse events, and (6) risk of bias.

### Risk of bias assessment

We assessed the risk of bias in the included studies using the Cochrane risk of bias (ROB 2) tool. Literature search, screening, and risk of bias assessment were carried out by two independent reviewers. Any disagreements or uncertainties were resolved through discussion and consensus between the reviewers. In cases where consensus could not be reached, a third reviewer was consulted for a final decision.

### Measures of treatment effect

The primary outcome for this systematic review and meta-analysis was the changes in hair count and diameter of CMNT versus minoxidil monotherapy while focusing on the impact of microneedling parameters on hair count, specifically depth (≤ 1 mm vs. >1 mm), duration (≤ 12 weeks vs. >12 weeks), and technique (electrodynamic vs. rolling). These outcomes were evaluated at both baseline and post-treatment. The treatment effects were measured using the mean difference (MD) and standardized mean difference (SMD), with corresponding 95% confidence intervals (CIs). Other outcomes included investigator’s assessment scores and patient’s self-assessment scores, which were rated using a standardized 7-point scale (–3 = Severe worsening, − 2 = Moderate worsening, − 1 = Mild worsening, 0 = no change, + 1 = Mild improvement, + 2 = Moderate improvement, + 3 = Excellent improvement) [19].For the meta-analysis, scores of − 3, − 2, and − 1 were grouped into the “Worsened” category, while scores of + 1, +2, and + 3 were combined into the “Improved” category. The effect estimates for these outcomes were reported as an odds ratio with 95% confidence interval (CI) for “Improved,” “No Change,” and “Worsened” groups. Lastly, safety outcome was reported as the number of patients experiencing adverse events in both the CMNT and minoxidil monotherapy groups.

### Data synthesis

All statistical analyses were conducted using R version 4.4. All data was reported as counts and frequencies for qualitative variables and as means and standard deviation for quantitative variables. Effect sizes for continuous outcomes, such as the change in hair count and hair diameter, were expressed as SMD with 95% confidence intervals (CIs). For dichotomous outcomes, such as investigator’s and patient’s assessments of hair condition, odds ratios (OR) with 95% CIs were used. In the analysis of adverse events, risk ratios (RRs) with 95% CIs were reported. Statistical significance was set at a p-value < 0.05. A random-effect model was applied due to the variations in patient demographics and MN parameters, such as microneedling depth, intervention duration, and technique. A funnel plot was conducted for the mean difference in hair count between CMNT and minoxidil monotherapy to assess any publication bias. We performed a leave-one-out analysis by systematically removing one study at a time to evaluate the influence of each individual study on the overall effect size and heterogeneity.

### Assessment of heterogeneity

We used Cochran Q test (chi-square test) and Higgins and Thompson (I^2^) to assess the heterogeneity among the included studies via the following equation: *I*^*2*^*= ((Q-df)/Q) ×100%.* Heterogeneity was considered significant when the chi-square test p-value is less than 0.05 and the I^2^ test is greater than 50% hi [20]. Statistically significant heterogeneity was addressed by switching to the random-effect model.

## Result

### Characteristics of the included studies

A total of **631** AGA subjects, aged 18–60 years, were enrolled from 12 RCTs including one split scalp study [21]. The severity of hair loss was Hamilton Norwood II-VI for male subjects and Ludwig I-III for female subjects. The studies primarily involved two intervention groups: those receiving CMNT (335 participants) and those receiving minoxidil alone (315 participants). The baseline characteristics of participants are summarized in Table 1.

**Table 1:** The characteristics of the included studies

Study	Country	RCT Type	Total Participants	Age		% Male	AGA Scale	
				Mean	(Range)		Hamilton-Norwood	Ludwing (female)
							(male)	
Dhurat [39]	India	Single-blinded, parallel, two-arms	100	28.6	(20-35)	100%	III vertex-IV	N/A
Yu [21]	China	Single-blinded, parallel, two-arms	19	35.2 ± 6.8	(23-45)	100%	III-VI	N/A
Kumar [40]	India	Single-blinded, parallel, two-arms	68	N/A	(18-40)	100%	III-IV	N/A
Bao [27]	China	Single-blinded, parallel, three-arms	60	N/A	(20-50)	100%	III-VI	N/A
Faghihi [26]	Iran	Single-blinded, parallel, three-arms	60	30.32 ±	(18-45)	51%	III-VI	Types 2, 3
				7.06				
Malhotra & Herakal [29]	India	Non-blinded, parallel,	60	N/A	(21-40)	100%	III-IV	N/A
		two-arms						
Sohng [23]	Korea	Non-blinded, parallel,	29	N/A	(31-54)	83%	II-V	Type 1
		three-arms						
Bao [30]	China	Non-blinded, parallel,	75	N/A	(20-60)	100%	III-VI	N/A
		three-arms						
Liang [25]	China	Single-blinded, parallel, three-arms	120	N/A	(18-45)	0%	N/A	Types 2, 3
Zhang [28]	China	Single-blinded, parallel, two-arms	40	30.87 ±	(18-50)	0%	N/A	N/A
				5.2				
Adistri [24]	Indonesia	Non-blinded, parallel,	36	34 ± 6.75	(26-51)	100%	III-VI	N/A
		two-arms						
Yasmeen & Haque [22]	Bangladesh	Non-blinded, parallel,	90	29.90 ±	(18-45)	50%	II-IV	N/A
		three-arms		5.5				

The administration of topical minoxidil was largely consistent across studies, with most studies utilizing 5% minoxidil applied twice daily, while there were variations in preparation and delivery techniques of microneedling therapy. The detailed characteristics of minoxidil (M) and (CMNT) are presented in Table 2.

**Table 2:** Treatment protocol of the included studies

Study	Minoxidil Group (M)	M Characteristics		CMNT Group	MN Characteristics			Duration of treatment (weeks)
		M%	M Frequency		MN Type	MN Depth (mm)	MN Interval (week)	
Dhurat [39]	M (44)	5%	Twice daily (BID)	MN+M (50)	Rolling	1.5	Once / Week	12
Yu [21]	M (19)	5%	Twice daily (BID)	MN+M (19)	Fractional Radiofrequency MN (FRM)		Once / 4 Weeks	20
						1.5		
Kumar [40]	M (29)	5%	Twice daily (BID)	MN+M (31)			Once / Week	12
					Rolling	1.5		
Bao [27]	M (18)	5%	Twice daily (BID)	MN+M (20)	Electrodynamic	1.5-2.5	Once / 2 Weeks	24
Faghihi [26]	M (20)	5%	Twice daily (BID)	MN+M (39)	Electrodynamic	0.6,1.2	Once / 2 Weeks	12
Malhotra & Herakal [29]	M (30)	5%	Twice daily (BID)	MN+M (30)	Rolling		Once / 2 Weeks	24
						1.5		
Sohng [23]	M (9)	5%	N/A	MN+M (9)	Spiral grooved MN		Twice / Week	26
						0.25		
Bao [30]	M (23)	5%	Twice daily (BID)	MN+M (25)	Electrodynamic	01-Feb	Once / 3 Weeks	24
Liang [25]	M (38)	5%	Once daily	MN+M (40)	Electrodynamic	0.7-1	Once / 2 Weeks	24
Zhang [28]	M (20)	2%	Twice daily (BID)	MN+M (20)	Nano-device: 3DL-GG: Nanomed Device Inc.	0.26	Once / Week	24
Adistri [24]	M (18)	5%	Twice daily (BID)	MN+M (18)	Rolling	0.6	Once / 4 Weeks	12
Yasmeen & Haque [22]	M (30)	5%	Twice daily (BID)	MN+M (30)	Rolling	1.5	Once / 4 Weeks	20

### Risk of bias in included trials

**Fig. 2:**
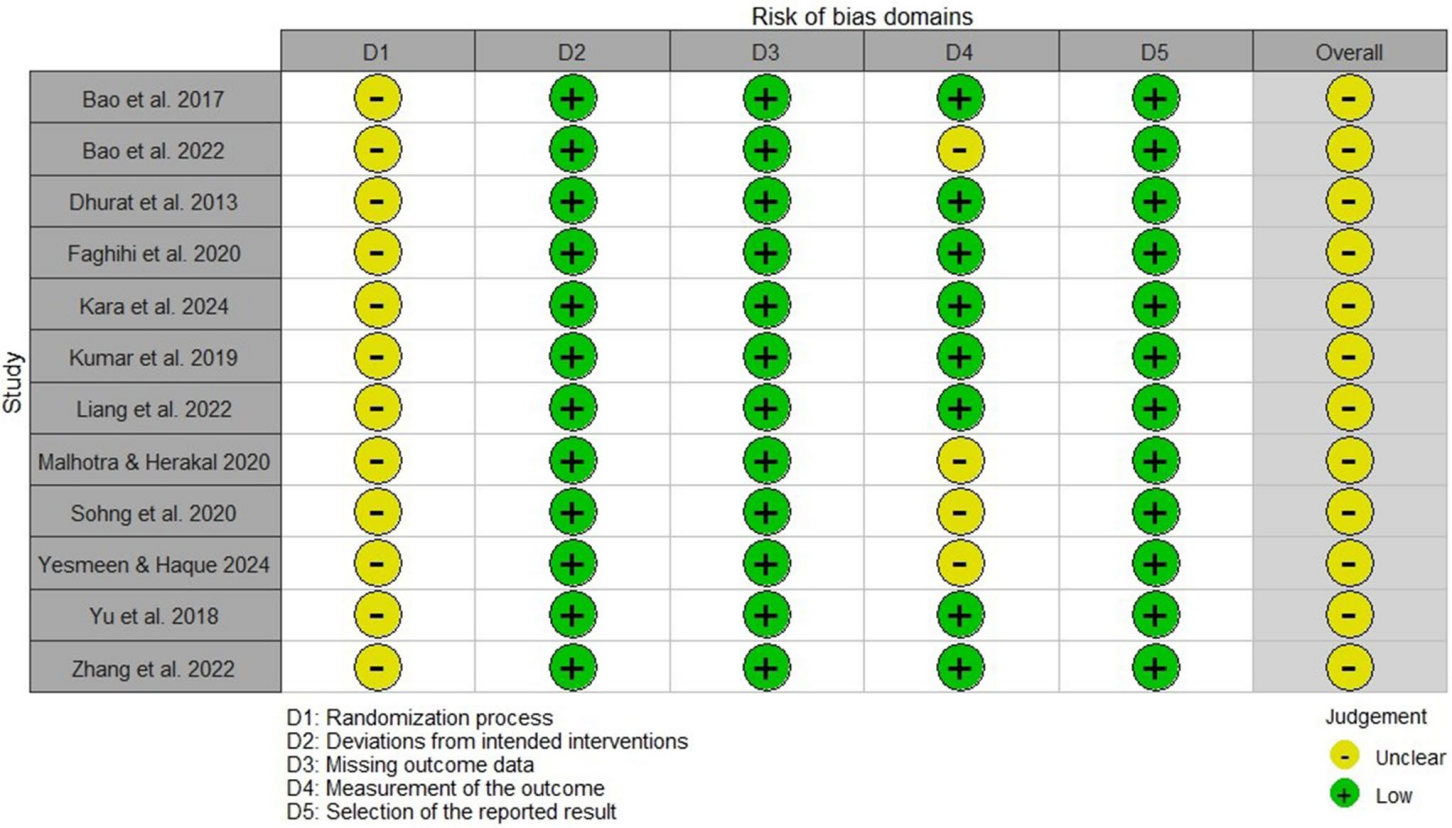
Traffic light plot summarizing the risk of bias assessment for the included studies

**Fig. 3:**
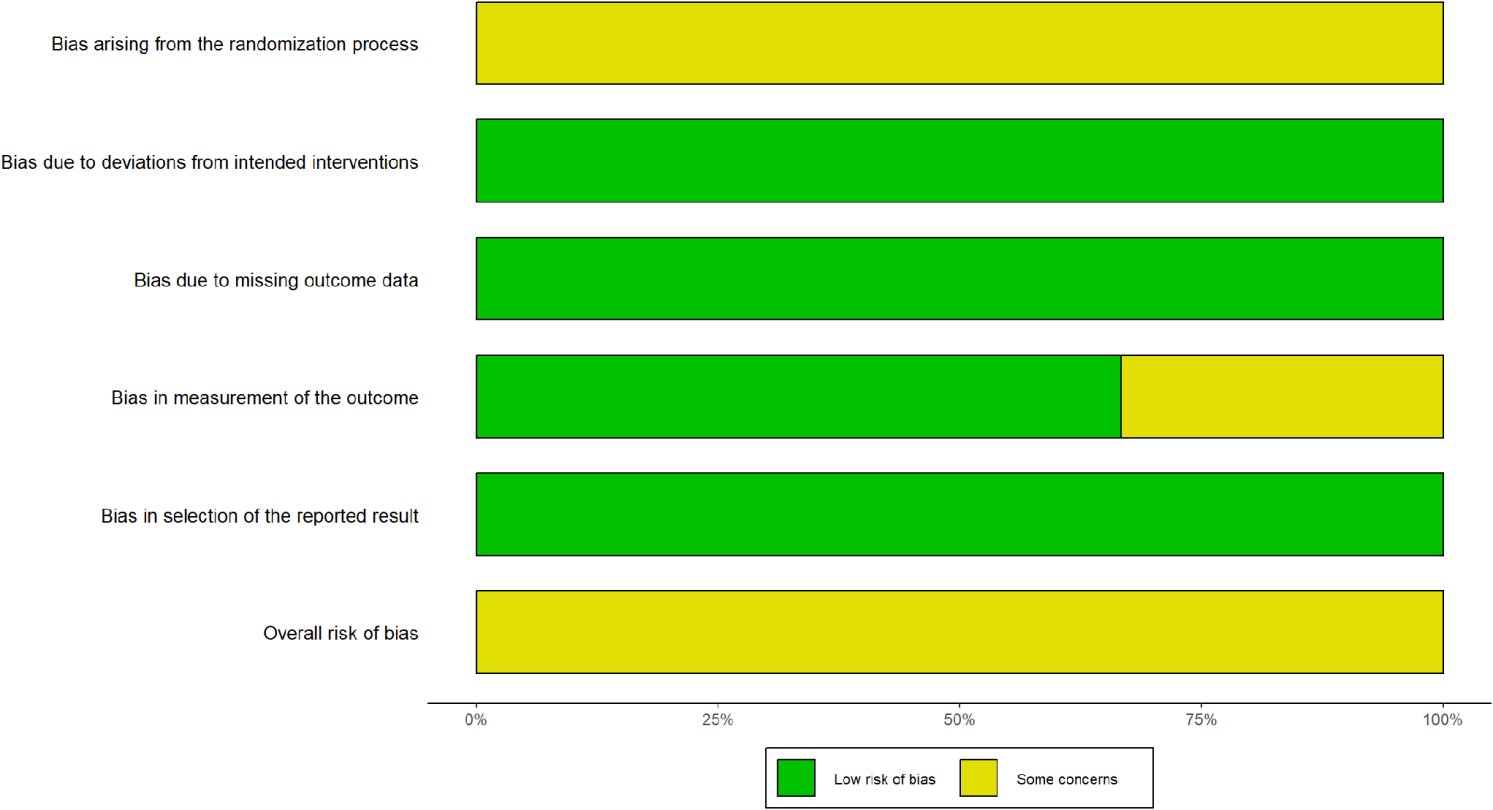
Weighted bar plots illustrating the distribution of risk-of-bias judgments within each bias domain, categorizing them into low, some concern, or high risk

Figure 2 provides a detailed risk of bias assessment for the included studies. The overall risk of bias assessment revealed that all included studies had a low risk of bias in domains related to deviations from intended interventions, missing outcome data, and selection of reported results. However, concerns were identified regarding the randomization process across all studies and the measurement of outcomes in some studies, particularly where patients’ self-assessments were reported [22, 23]. Overall, all studies were considered as having “some concern”. The overall risk of bias for each domain is presented in Fig. 3.

### Efficacy

#### Changes in hair count

**Fig. 4:**
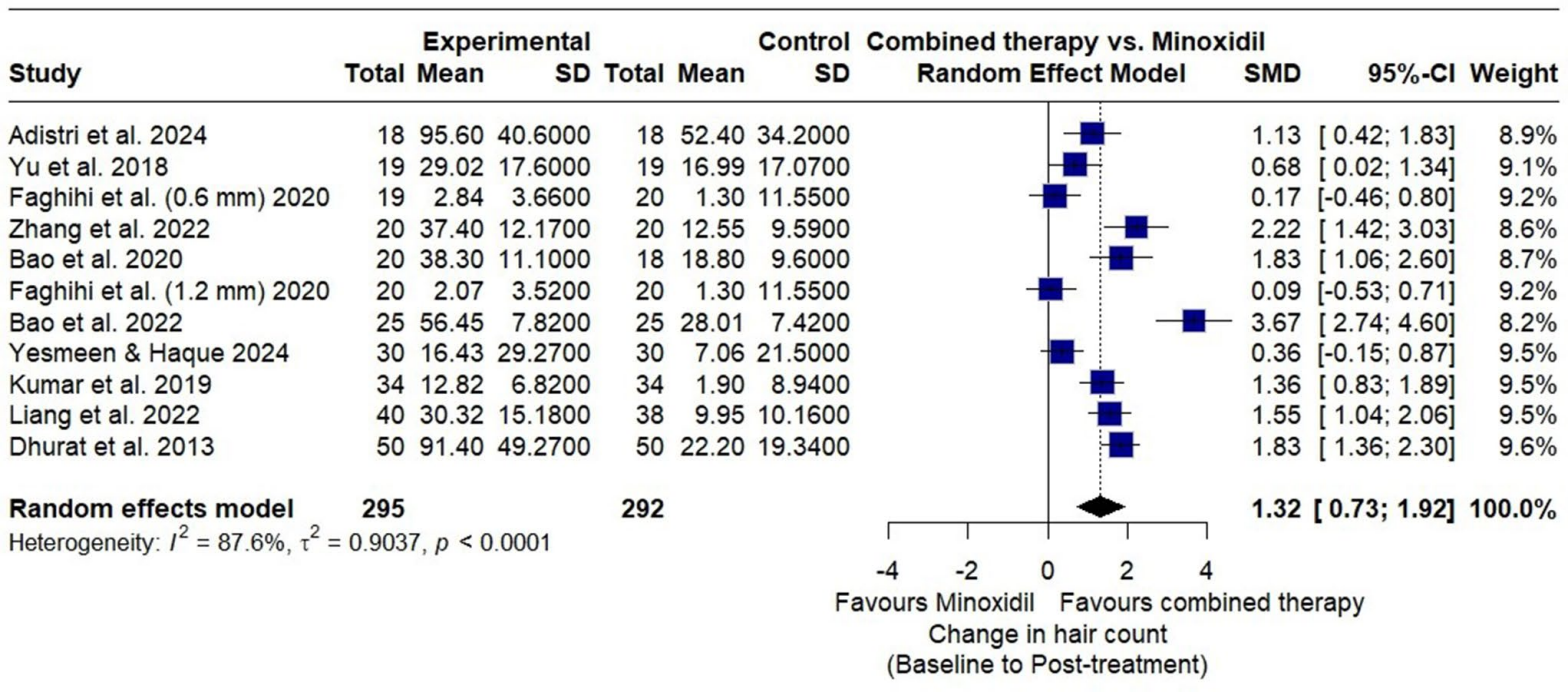
Forest plot showing the overall standardized mean difference (SMD) of hair count between the combined microneedling and minoxidil group versus the minoxidil monotherapy group

Data on the mean difference (MD) in total hair count was collected from ten RCTs with 587 participants (295 participants in the combined microneedling therapy and 292 in the minoxidil monotherapy). The overall SMD of change in hair count, estimated using a random effect model, favored the combined microneedling with minoxidil therapy over minoxidil monotherapy alone. (SMD 1.32, 95% CI 0.73 to 1.92; *p* < 0.01). There was substantial heterogeneity among the included studies (I² = 88%; *p* < 0.01) (Fig. 4). The leave-one-out analysis revealed no significant differences in the overall effect size or heterogeneity.

**Fig. 5:**
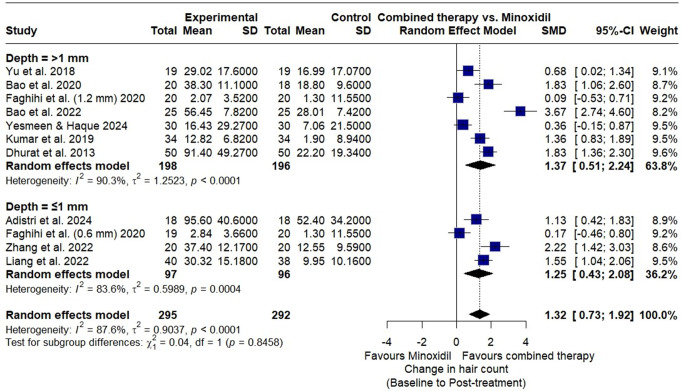
Forest plot of subgroup analysis demonstrating the effect of microneedling depth (> 1 mm vs. ≤1 mm) combined with minoxidil versus minoxidil monotherapy on hair count

A subgroup analysis assessed microneedling depth (> 1 mm vs. ≤1 mm) on hair count improvement. Seven RCTs with depths > 1 mm showed a pooled SMD of 1.37 (95% CI: 0.51–2.24), favoring combined therapy with substantial heterogeneity (I² = 90%; *p* < 0.01). Four RCTs with depths ≤ 1 mm yielded a pooled SMD of 1.25 (95% CI: 0.43–2.08), also favoring the combination with high heterogeneity (I² = 84%; *p* < 0.01). Subgroup differences were not significant (χ² = 0.04, df = 1; *p* = 0.85) (Fig. 5).

**Fig. 6:**
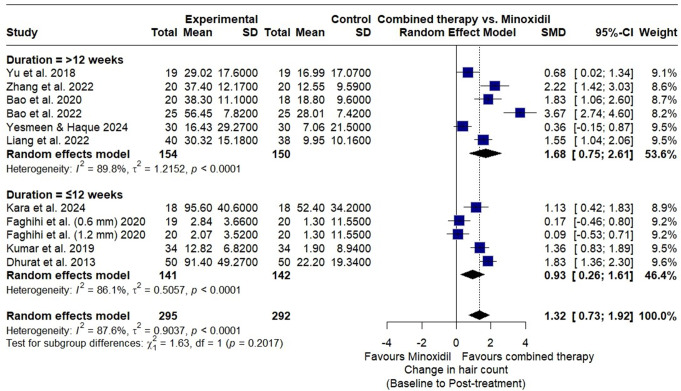
Forest plot of subgroup analysis illustrating the effect of microneedling therapy duration (> 12 weeks vs. ≤12 weeks) combined with minoxidil versus minoxidil monotherapy on hair count

A subgroup analysis compared microneedling therapy duration (> 12 weeks vs. ≤12 weeks) on hair count improvement with minoxidil. For > 12 weeks, pooled SMD was 1.68 (95% CI: 0.75–2.61) with substantial heterogeneity (I² = 90%; *p* < 0.01). For ≤ 12 weeks, SMD was 0.93 (95% CI: 0.26–1.61) with similar heterogeneity (I² = 86%; *p* < 0.01). Subgroup differences were not significant (χ² = 1.63, df = 1; *p* = 0.20) (Fig. 6).

**Fig. 7:**
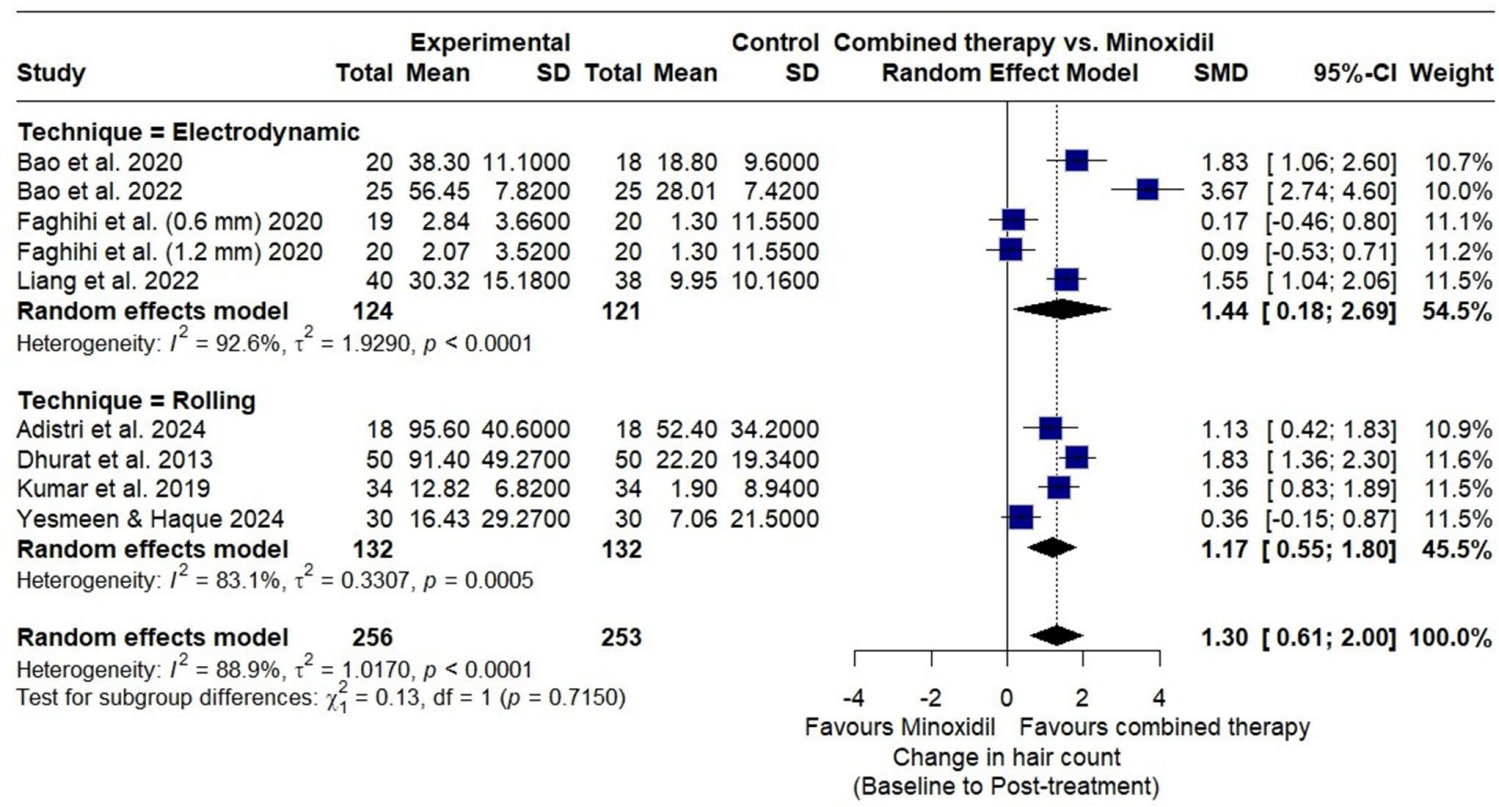
Forest plot of subgroup analysis examining the impact of microneedling technique (device) (electrodynamic vs. rolling) combined with minoxidil versus minoxidil monotherapy on hair count

A subgroup analysis based on microneedling technique showed significant hair count improvements with both rolling and electrodynamic microneedling combined with minoxidil. Electrodynamic microneedling had a slightly higher SMD (1.44; 95% CI: 0.18–2.69) than rolling (SMD = 1.17; 95% CI: 0.55–1.80). High heterogeneity was observed (I² = 93% for electrodynamic, I² = 83% for rolling). Subgroup differences were not significant (χ² = 0.13, df = 1; *p* = 0.72) (Fig. 7).

A meta-regression was conducted to assess whether study-level factors, including sample size of the intervention and control groups, participant age, and follow-up duration, influenced the effect size for changes in hair count (Table 3). The results indicate that none of the variables tested had a statistically significant impact on the pooled effect size. Specifically, the sample size of the intervention group (estimate = -0.219; *p* = 0.62), the control group (estimate = 0.2747; *p* = 0.54), participant age (estimate = 0.1547; *p* = 0.26), and follow-up duration (estimate = 0.0852; *p* = 0.26) were not significant predictors of the observed heterogeneity (I² = 88%; *p* < 0.01). These findings suggest that the substantial heterogeneity cannot be explained by these study-level factors, implying that other, unmeasured variables or individual-level factors may be contributing to the variability in effect sizes.

**Table 3:** Meta regression analysis

Model	Estimate	SE	z-value	*p*-value	95% C.I.	
Intercept	-6.4954	4.6528	-1.396	0.1627	-15.6147	2.6239
Sample size (Intervention)	-0.219	0.4355	-0.5029	0.6151	-1.0727	0.6346
Sample size (Control)	0.2747	0.4471	0.6144	0.539	-0.6016	1.151
Age	0.1547	0.1385	1.1168	0.2641	-0.1168	0.4262
Follow-up	0.0852	0.0759	1.1231	0.2614	-0.0635	0.234

#### Change in hair diameter

**Fig. 8:**
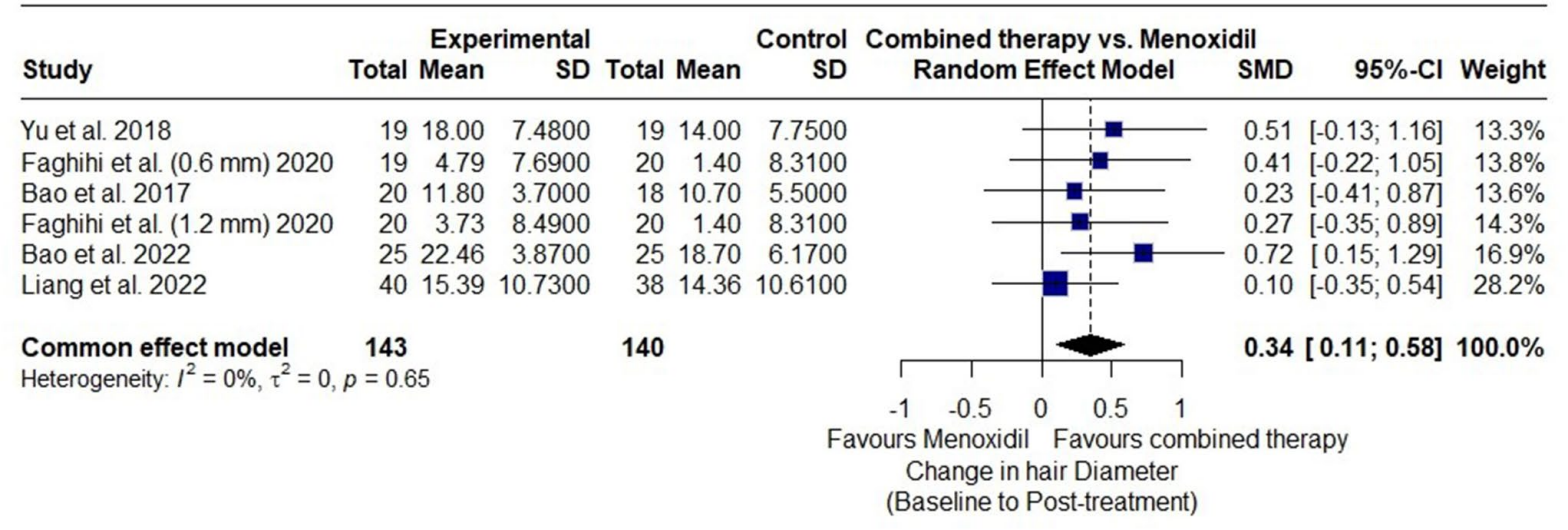
Forest plot showing the overall standardized mean difference (SMD) of hair diameter between the combined microneedling and minoxidil group and the minoxidil monotherapy group

Six RCTs assessed hair diameter at baseline and post treatment, including 283 participants, (143 participants in the combined microneedling and minoxidil group and 140 participants in minoxidil monotherapy group). The overall SMD 0.34 (95% CI: 0.11 to 0.58; *p* < 0.01) favored the combined microneedling and minoxidil therapy over the minoxidil monotherapy. There is no observed heterogeneity among the studies (I² = 0%; *p* = 0.65). The overall results demonstrated a statistically significant improvement in hair diameter with combined therapy (Fig. 8).

#### Investigator’s assessment score

**Fig. 9:**
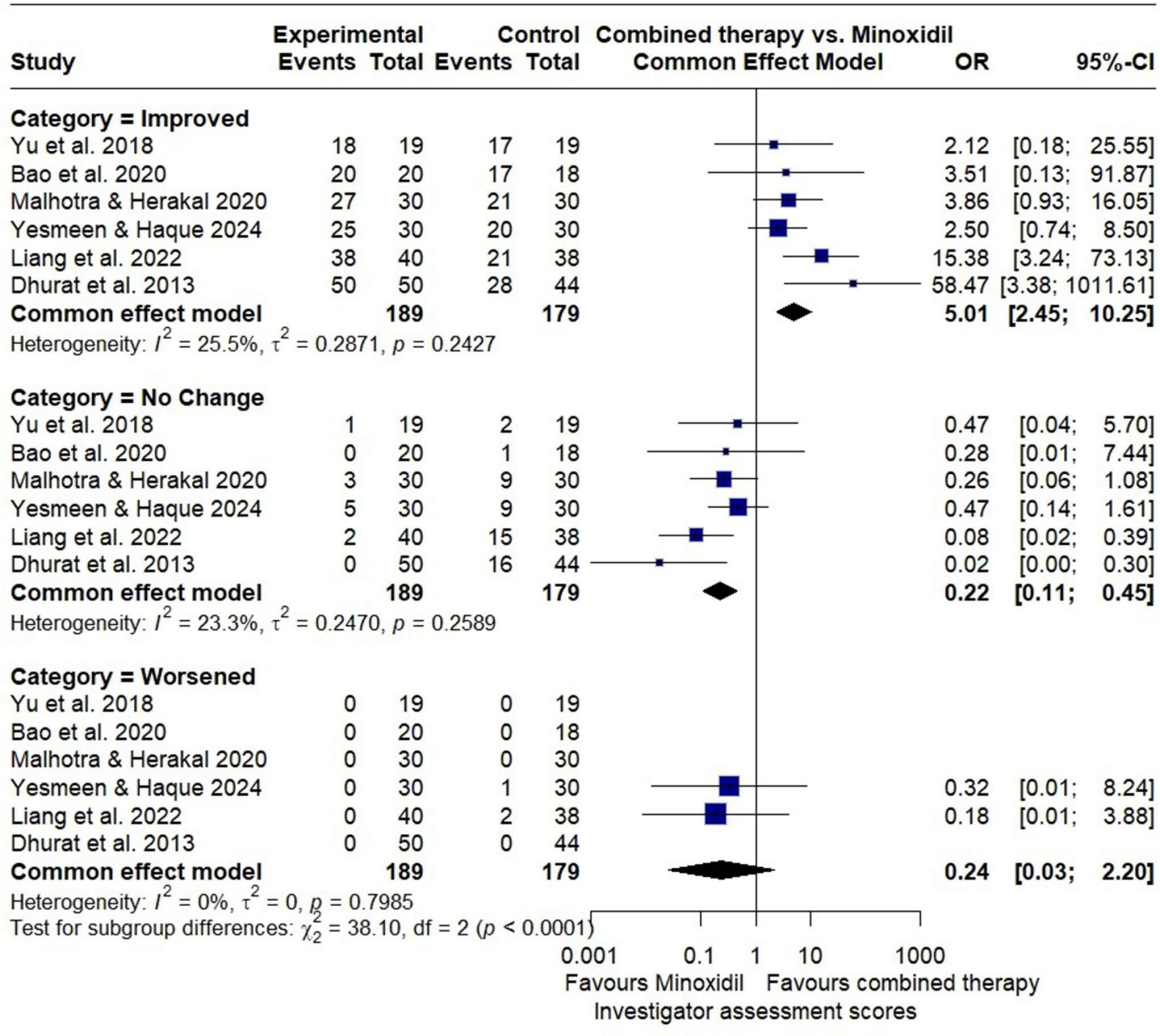
Forest plot of odds ratios comparing investigator’s assessment categories (improved, no change, and worsened) between the combined microneedling and minoxidil group and the minoxidil monotherapy group

Six RCTs reported the investigator’s assessment scores of hair improvement with a total of 368 patients assessed. The combined microneedling therapy with minoxidil showed significantly better odds of improvement compared to minoxidil alone (OR = 5.01; 95% CI: 2.45 to 10.25) with (I² = 26%).Additionally, the odds of no change were reduced in the combined therapy group (OR = 0.22; 95% CI: 0.11 to 0.45) with (I² = 23.3%), and the odds of worsening were also lower (OR = 0.24; 95% CI: 0.03 to 2.20) with no observed heterogeneity (Fig. 9).

#### Patient’s self-assessment score

**Fig. 10:**
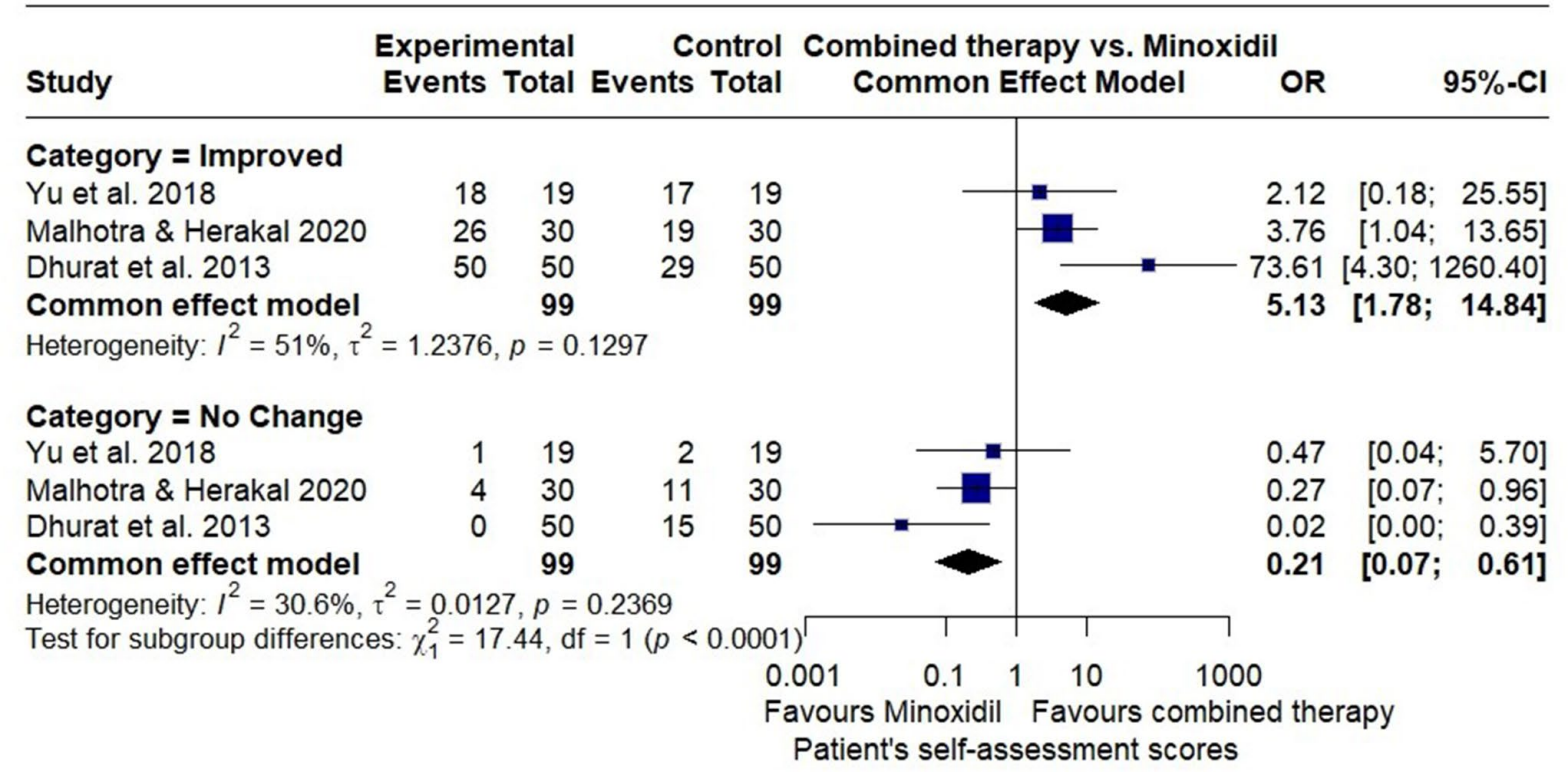
Forest plot of odds ratios comparing patient’s self-assessment score categories (improved, no change, and worsened) between the combined microneedling and minoxidil group and the minoxidil monotherapy group

Three RCTs reported a patient’s self-assessment score. The pooled OR for improvement with combined therapy was 5.13 (95% CI; 1.78 to 14.84), while the group using minoxidil monotherapy were more likely to report no change in hair condition (OR = 0.21; 95% CI, 0.07 to 0.61), favoring combined therapy (Fig. 10).

### Safety (adverse events)

**Fig. 11:**
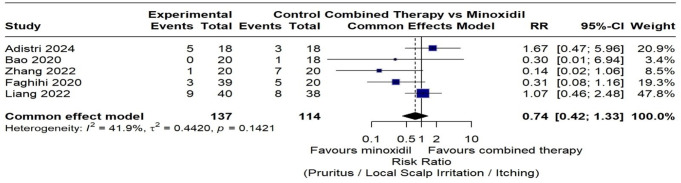
Forest plot of relative risk of scalp itching in the combined microneedling therapy compared to minoxidil monotherapy group

Seven of the included studies reports the adverse event outcome [24–30]. Adverse events were more frequent in the CMNT group (74 events) compared to the minoxidil group (59 events). Scalp itching was the most frequently reported adverse event in both treatment groups, with a higher incidence observed in the minoxidil monotherapy group. However, a meta-analysis of five RCTs assessing scalp itching as an adverse event found no statistically significant difference in the risk of scalp itching between CMNT and minoxidil monotherapy (Risk Ratio (RR) = 0.74, 95% CI: 0.42–1.33, *p* = 0.32) with (I² = 41.9%) (Fig. 11).

**Fig. 12:**
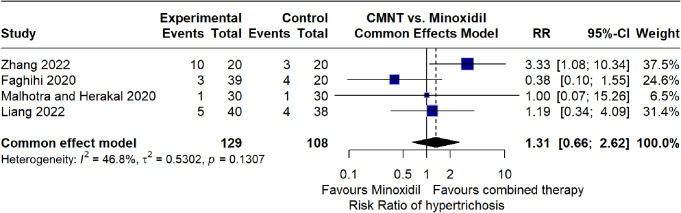
Forest plot of relative risk (RR) of hypertrichosis in the combined microneedling therapy compared to minoxidil monotherapy group

Hypertrichosis was more frequently observed in the combined microneedling therapy (CMNT) group. However, a meta-analysis of four RCTs assessing hypertrichosis as an adverse event found no statistically significant difference in its risk between CMNT and minoxidil monotherapy (RR = 1.31, 95% CI: 0.66–2.62, *p* = 0.44) with (I² = 46.8%) (Fig. 12).

Eczema, urticaria, and palpitations were reported only in the minoxidil group, whereas enlarged lymph nodes, dandruff, and infection were observed exclusively in the CMNT group. Details of adverse events are summarized in Table 4.

**Table 4:** Adverse events reported in CMNT group and Minoxidil monotherapy

Adverse event	M Group	CMNT Group
Headache	4 [25, 29]	6 [28, 29]
Hypertrichosis	12 [25, 26, 28, 29]	19 [25, 26, 28, 29]
Scalp itching	25 [24, 27, 28, 29]	18 [24, 28]
Seborrheic dermatitis	2 [27, 30]	2 [27, 30]
Eczema	2 [27, 30]	0
Enlarged lymph nodes	0	9 [26, 27, 30]
Increased scurf	7 [25]	6 [25, 27]
Discomfort over the scalp	1 [29]	3 [25]
Contact dermatitis	1 [25]	1 [25]
Erythema	2 [25]	7 [24, 29]
Dandruff	7 [25]	7 [25, 27, 30]
Palpitation	1 [25]	0
Postural hypotension	1 [25]	1 [25]
Urticaria	1 [25]	0
Infection	0	1 [25]
Total	59	74

### Additional outcomes

Five studies [21, 24, 25, 27, 30]have also reported additional outcomes related to hair growth. However, due to the limited number of studies reporting these outcome indicators, a meta-analysis could not be performed. Instead, a table has been created to present the results (Table 5).

**Table 5:** Additional outcomes reported in the included studies

Study ID	Outcome	Result
Bao [27]	VAS pain score	The mean VAS score for participants who received MN therapy was 4.52 ± 3.7.
Bao [30]	Molecule expression in the Wnt/β-catenin signaling pathway	There was a statistically significant difference (*p* < 0.05) in the upregulation of FZD3, β-catenin, and LEF-1 expression at both the mRNA and protein levels in the treated areas of the MN + M group.
Yu [21]	VAS pain score	The mean VAS score for all participants treated with FRM therapy was 3.63 ± 1.38.
Liang [25]	Change in scalp tissue structure	The epidermal thickness was significantly increased only in MN + M group (*p* < 0.001) The dermis thickness was significantly increased in two groups (*p* < 0.001) The average follicle diameter was significantly increased only in MN + M group (*p* < 0.001)
Adistri [24]	Changes in terminal and vellus hair	After 12 weeks, the MN + M group had a higher percentage of terminal hair (73.2%) compared to the M group (58.8%), while the vellus hair percentage was lower in the MN + M group (26.8%) compared to the M group (41.1%).

### Publication bias

**Fig. 13:**
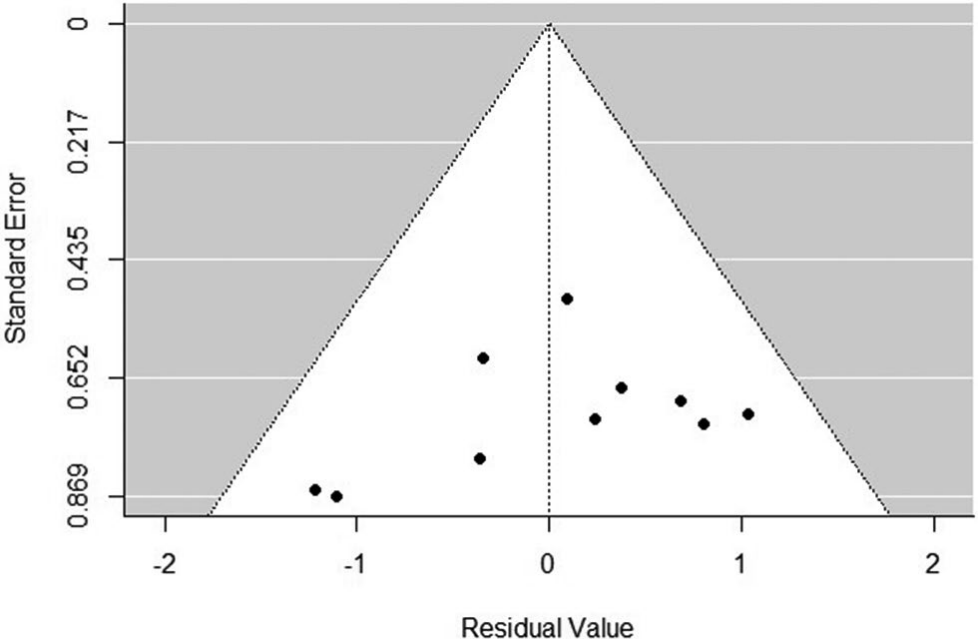
Funnel plot assessing potential publication bias for the hair count outcome across the included studies

A funnel plot for the mean difference of the hair count between CMNT and minoxidil monotherapy demonstrated a relative symmetry with most studies fall within the 95% confidence intervals. However, a slight asymmetry is observed, which may reflect small-study effects or methodological heterogeneity rather than true publication bias (Fig. 13).

## Discussion

### Meta-analysis of hair count

In this study, we observed a significant improvement in hair count in CMNT with minoxidil compared to minoxidil monotherapy. However, substantial heterogeneity was present. Our results align with those of Abdi et al., who reported a pooled mean difference of 1.76 (95% CI: 1.26 to 2.26) across eight studies, also with significant heterogeneity [31].Xu et al., on the other hand, found a higher mean difference 15.82 (95% CI: 12.34 to 19.31) with no significant heterogeneity across seven studies [32]. A key difference is that Xu et al.’s analysis mainly included studies from China with smaller sample sizes which may have led to greater consistency in protocols and participant demographics and might have reduced heterogeneity [32].

Further attempts to explain the effect of MN parameters and understand heterogeneity involved subgroup analyses based on factors potentially impacting the hair count outcome such as needle depth (> 1 mm vs. ≤1 mm), treatment duration (> 12 weeks vs. ≤12 weeks), and device type (rolling vs. electrodynamic). These subgroup analyses showed that none of these parameters affected the hair count. Our results contradict those of Xu et al., who suggested that a 12-week treatment period was more effective than 21–24 weeks [32]. While Lima et al. demonstrated that roller devices with longer needles (e.g., 3 mm) achieved actual penetration depths of approximately 1.5–2.0 mm, influenced by factors such as user pressure and needle angulation [32], Sasaki et al. observed that derma pen devices achieved penetration depths closely matching their needle lengths (0.25–1 mm), attributed to their perpendicular needle entry and automated mechanism [33]. Faghihi et al. who also used an electrical MN device, reported that shorter needles (0.60 mm) used biweekly in combination with minoxidil resulted in greater improvements in hair count and thickness compared to longer needles (1.20 mm) [26]. In contrast, our findings indicated that both device types (roller and pen) were similarly effective when combined with minoxidil, and that a higher microneedling depth than 1 mm depth did not significantly increase hair count.

### Meta-analysis of hair diameter

The pooled analysis of the six identified RCTs studying hair diameter showed a significant improvement in hair diameter with CMNT with minoxidil compared to minoxidil monotherapy, with no observed heterogeneity. In contrast, the systematic review by Abdi et al. did not show a significant increase in hair diameter with substantial heterogeneity among the included studies [31].The mechanisms underlying microneedling further support our findings, as it stimulates growth factors such as vascular endothelial growth factor (VEGF) and platelet-derived growth factor (PDGF) enhances minoxidil penetration, and promotes collagen production, all of which contribute to increased hair thickness and follicle regeneration [34].

### Reported AEs

Scalp itching or pruritus was one of the common reported adverse events in both CMNT and minoxidil monotherapy groups, however; there was no statistically significant difference in the risk of itching between both groups. According to Friedman et al., itching is primarily associated with irritant or allergic contact dermatitis, often triggered by minoxidil solution ingredients such as propylene glycol, or by the exacerbation of pre-existing seborrheic dermatitis [35].Although microneedling may cause mechanical disruption of the epidermis, our study showed that itching was more prevalent with minoxidil monotherapy compared to CMNT. This might suggest that microneedling does not exacerbate pruritus when combined with minoxidil. While microneedling (MN) is thought to improve the absorption of topical medications, such as minoxidil, our study did not find a significant difference in the occurrence of hypertrichosis in CMNT group and the minoxidil monotherapy group. Further larger sample size research is necessary to explore whether MN also significantly increases the occurrence of hypertrichosis and other minoxidil reported side effects when used as conjugation therapy.

### Strengths

Following PRISMA guidelines, a comprehensive search was conducted to include all relevant clinical trials on CMNT therapy for AGA. Our study provided a systemic review of a significant number of AGA patients (613 patients) across 12 RCTs. The use of standardized mean difference SMD as an effect measure allowed for the comparison of outcomes across studies that used different scales and measurements, ensuring consistency in data synthesis. In addition to evaluating objective indicators of hair growth, such as hair diameter and hair count, our study uniquely incorporated satisfaction surveys from both assessors and patients regarding hair growth outcomes. Importantly, our study focused on the impact of variations in microneedling parameters including treatment duration, depth, type of device, and on hair count improvement among a large and diverse population. Through a leave-one-out sensitivity analysis, we confirmed that the overall results remained stable, indicating that no single study disproportionately impacted the findings. Additionally, we conducted subgroup analyses based on microneedling depth, treatment duration, and device type, as well as a meta-regression to examine study-level factors such as sample size, participant age, and follow-up duration. The no heterogeneity observed in hair diameter outcomes suggests a consistent effect across studies, enhancing confidence in the reliability of these findings.

### Limitations

The findings of this systematic review should be considered with several limitations in mind. Firstly, the majority of the included RCTs were conducted in Asia, the Middle East, and North Africa, with no representation from Caucasian populations, where AGA is more prevalent [36].

Although our study included a substantial number of RCTs encompassing a broad age range of AGA patients, we were unable to perform detailed subgroup analyses by age. Such analyses could have offered valuable insights into whether treatment efficacy varies across age groups, especially given the progressive severity of AGA with advancing age [37, 38]. Although our research has explored the impact of MN duration on hair count, we observed variations in the intervals between sessions across different studies. Furthermore, all the included studies had some concern regarding the randomization process due to insufficient information about allocation sequence concealment.

Future research is necessary to determine treatment efficacy across different populations and age groups. Additional efforts are required to ascertain the optimal number of sessions needed to achieve the best treatment outcomes while avoiding side effects from prolonged MN application.

## Conclusion

This systematic review and meta-analysis suggest that microneedling when combined with minoxidil significantly improve hair count and hair diameter in AGA patients. This review also suggests variations in microneedling parameters such as depth (≤ 1 mm vs. >1 mm) treatment duration (≤ 12 weeks vs. >12 weeks), or technique(device) (electrodynamic vs. rolling) may not significantly enhance hair count in these patients.

## Electronic supplementary material

Below is the link to the electronic supplementary material.

Supplementary Material 1

## Data Availability

No datasets were generated or analysed during the current study.
