# Supplementary material for: Evaluating the efficacy and safety of combined microneedling therapy versus topical Minoxidil in androgenetic alopecia: a systematic review and meta-analysis
Source: Arch Dermatol Res. 2025 Mar 8;317(1):528. doi: 10.1007/s00403-025-04032-1 (PMC11890238; doi:10.1007/s00403-025-04032-1)
Supplement: Supplementary file 1 — Supplementary Material 1 [file 403_2025_4032_MOESM1_ESM.docx]

**Search strategy

PubMed**
("Microneedle"[MeSH] OR Microneedle OR MN OR "Percutaneous Collagen Induction" OR Needling)

AND ("Androgenic Alopecia"[MeSH] OR "Androgenic Alopecia" OR "Male Pattern Baldness" OR "Female Pattern Baldness" OR AGA)

AND ("Combined Therapy" OR Minoxidil OR PRP OR "Platelet-Rich Plasma" OR "Fibroblast Growth Factor" OR FGF)
 **Scopus**
TITLE-ABS-KEY("Microneedle" OR MN OR "Percutaneous Collagen Induction" OR Needling)

AND TITLE-ABS-KEY("Androgenic Alopecia" OR "Male Pattern Baldness" OR "Female Pattern Baldness" OR AGA)

AND TITLE-ABS-KEY("Combined Therapy" OR Minoxidil OR PRP OR "Platelet-Rich Plasma" OR "Fibroblast Growth Factor" OR FGF) **Web of science**TS=("Microneedle" OR MN OR "Percutaneous Collagen Induction" OR Needling)

AND TS=("Androgenic Alopecia" OR "Male Pattern Baldness" OR "Female Pattern Baldness" OR AGA)

AND TS=("Combined Therapy" OR Minoxidil OR PRP OR "Platelet-Rich Plasma" OR "Fibroblast Growth Factor" OR FGF)

**Google Scholar**"Microneedle" OR "MN" OR "Percutaneous Collagen Induction" OR "Needling"

AND "Androgenic Alopecia" OR "Male Pattern Baldness" OR "Female Pattern Baldness" OR "AGA"

AND "Combined Therapy" OR "Minoxidil" OR "PRP" OR "Platelet-Rich Plasma" OR "Fibroblast Growth Factor"

**Cochrane Library**("Microneedle" OR MN OR "Percutaneous Collagen Induction" OR Needling) AND ("Androgenic Alopecia" OR "Male Pattern Baldness" OR "Female Pattern Baldness" OR AGA) AND ("Combined Therapy" OR Minoxidil OR PRP OR "Platelet-Rich Plasma" OR "Fibroblast Growth Factor" OR FGF) **Embase**('microneedle' OR 'MN' OR 'percutaneous collagen induction' OR 'needling') AND ('androgenic alopecia' OR 'male pattern baldness' OR 'female pattern baldness' OR 'AGA') AND ('combined therapy' OR 'minoxidil' OR 'PRP' OR 'platelet-rich plasma' OR 'fibroblast growth factor' OR 'FGF')
